# Molecular Characterization of Prostate Cancers in the Precision Medicine Era

**DOI:** 10.3390/cancers13194771

**Published:** 2021-09-24

**Authors:** Emilio Francesco Giunta, Laura Annaratone, Enrico Bollito, Francesco Porpiglia, Matteo Cereda, Giuseppe Luigi Banna, Alessandra Mosca, Caterina Marchiò, Pasquale Rescigno

**Affiliations:** 1 Medical Oncology, Department of Precision Medicine, Università degli Studi della Campania “Luigi Vanvitelli”, 80131 Naples, Italy; emiliofrancescogiunta@gmail.com; 2 Department of Medical Sciences, University of Turin, 10126 Turin, Italy; laura.annaratone@ircc.it (L.A.); caterina.marchio@unito.it (C.M.); 3 Pathology Unit, Candiolo Cancer Institute, FPO-IRCCS, Candiolo, 10060 Turin, Italy; 4 Department of Pathology, University of Turin, San Luigi Gonzaga Hospital, Orbassano, 10043 Turin, Italy; e.bollito@libero.it; 5 Department of Urology, University of Turin, San Luigi Gonzaga Hospital, Orbassano, 10043 Turin, Italy; francesco.porpiglia@unito.it; 6 Cancer Genomics and Bioinformatics Unit, IIGM-Italian Institute for Genomic Medicine, c/o IRCCS Candiolo, 10060 Turin, Italy; matteo.cereda@iigm.it; 7 Candiolo Cancer Institute, FPO—IRCCS, Str. Prov.le 142, km 3.95, 10060 Candiolo, Italy; 8 Department of Oncology, Portsmouth Hospitals University NHS Trust, Portsmouth PO2 8QD, UK; gbanna@yahoo.com; 9 Multidisciplinary Outpatient Oncology Clinic, Candiolo Cancer Institute, FPO-IRCCS, Candiolo, 10060 Turin, Italy; alessandra.mosca@ircc.it; 10 Interdisciplinary Group for Translational Research and Clinical Trials, Urological Cancers (GIRT-Uro), Candiolo Cancer Institute, FPO-IRCCS, Candiolo, 10060 Turin, Italy

**Keywords:** prostate cancer, precision medicine, predictive biomarkers, prognostic biomarkers, molecular oncology, liquid biopsy, PARP inhibitor, immunotherapy

## Abstract

**Simple Summary:**

Prostate cancer research has been recently characterized by the discovery of several prognostic and predictive molecular factors, which ultimately improve patients’ management. In this review, we present the clinical impact of such factors and the methods to detect them, both on tissue and blood, in advanced prostate cancer patients. The aim of this review is ultimately to depict the role of these molecular factors in the era of precision oncology.

**Abstract:**

Prostate cancer (PCa) therapy has been recently revolutionized by the approval of new therapeutic agents in the metastatic setting. However, the optimal therapeutic strategy in such patients should be individualized in the light of prognostic and predictive molecular factors, which have been recently studied: androgen receptor (AR) alterations, PTEN-PI3K-AKT pathway deregulation, homologous recombination deficiency (HRD), mismatch repair deficiency (MMRd), and tumor microenvironment (TME) modifications. In this review, we highlighted the clinical impact of prognostic and predictive molecular factors in PCa patients’ outcomes, identifying biologically distinct subtypes. We further analyzed the relevant methods to detect these factors, both on tissue, i.e., immunohistochemistry (IHC) and molecular tests, and blood, i.e., analysis of circulating tumor cells (CTCs) and circulating tumor DNA (ctDNA). Moreover, we discussed the main pros and cons of such techniques, depicting their present and future roles in PCa management, throughout the precision medicine era.

## 1. Introduction

Prostate cancer (PCa) is the second most common malignancy in men, after lung cancer, but with a relatively low mortality compared to other cancer types [1]. However, patients diagnosed with metastatic disease have a poor prognosis, with a 5-year survival of 30% [2].

Recent advances in both diagnosis and therapy have indeed prolonged survival of prostate cancer patients [3], especially in the light of approval of new drugs and a better knowledge of the underlying biological mechanisms for both castration-sensitive (CSPC) and castration-resistant (CRPC) prostate cancer patients.

Proper diagnosis and classification of PCa relies on robust histopathological examination, including cytological and architectural features that remain the cornerstone of PCa definition. Nevertheless, in the era of precision oncology, stratification of metastatic prostate cancer patients through molecular testing [4] has gained a prominent role, even in the attempt of tackling resistance to approved drugs, which remains a key issue and is ultimately responsible for patients’ death. Indeed, to date, the onset of castration resistance-which is an unavoidable event in the natural history of prostate cancer patients treated with androgen deprivation therapy (ADT) translates into poor survival [5,6].

In this review, we discuss the prognostic and predictive biomarkers in metastatic CRPC (mCRPC) and the methodologies currently used to identify them in both tissue and blood; moreover, we indicate pros and cons of these techniques according to distinct types of biological specimens, depicting the present and future of diagnostic workflow in this clinical scenario.

## 2. Predictive and Prognostic Markers

### 2.1. Androgen Receptor

Androgen receptor (AR) is a member of the steroid and nuclear receptor superfamily, acting as an intracellular transcriptional factor, and it is highly expressed in prostate cells [7]. Its main ligands are testosterone and 5α-dihydrotestosterone (5α-DHT), whose binding determines intracytoplasmic receptor activation, consisting of homodimerization, autophosphorylation, and its translocation to the nucleus [8]. AR gene, located on chromosome X (Xq12), encodes for a protein that has three main functional domains: N-terminal activation domain (NTD), central DNA binding domain (DBD), and C-terminal ligand binding domain (LBD) [9]. Between DBD and LBD, there is also the nuclear localization signal (NLS), responsible for translocation to the nucleus of the activated AR [10].

AR plays a fundamental role in prostate cancer development by ensuring cell survival and proliferation but also migration and invasion, which are hallmarks for human cancer [11]. Indeed, therapeutic approaches designed to suppress AR signaling in prostate cancer cells, mainly through inhibition of androgen biosynthesis by luteinizing hormone-releasing hormone (LHRH) agonist/antagonist, or through the use of receptor antagonists (antiandrogen drugs)—all these under the name of ADT—have been the main available weapons against metastatic prostate cancer for decades [12].

Disruptions of the AR pathway consist of AR point mutations, affecting both NTD and LBD, truncated variants, and gene amplifications, all of which confer selective advantage to prostate cancer cells with different mechanisms [13].

*AR* point mutations occur in the LBD encoding region (codons 665 to 920) [14] and are responsible for acquired resistance through alterations in the ligand’s affinity. Interestingly, these mutations exert their role in several ways: by reducing affinity to antiandrogenic drugs, such as flutamide and bicalutamide (V716T, W742C, and T878A mutation) or enzalutamide and apalutamide (F877L mutation), but also by modifying affinity for other endogenous-or exogenous-ligands such as a higher affinity for progesterone (H875Y and the aforementioned T878A mutation) and prednisone (L702H mutation) [15,16].

*AR* gene amplifications, which have been detected in up to 60% of pretreated CRPC patients [17], are also responsible for tumor progression despite optimal ADT; in fact, they determine higher expression of AR in prostate cancer tissue with consequent cell growth despite low androgen levels [18]. Gene amplifications are rarely detected in treatment-naïve patients, suggesting they have a role in adaptive response to antiandrogen therapies [13].

AR variants consist in protein transcriptions characterized by LBD loss with a different length of C-terminal domain, determining constitutively activated truncated ARs that translocate to the nucleus [19]. These variants, named AR-V7 (also known as AR3) [20], AR-V12 (also known as ARv567es) [21], and AR-V3, have been found in CRPC patients, and among them, AR-V7 is the most frequent alteration detected—up to 75% of CRPC on ADT [22]. In the same setting of patients, a recent work has highlighted the possibility of co-occurrence of some of these variants [23].

Alterations of AR are indeed rare in treatment-naïve metastatic prostate cancer, probably being random “passenger” mutations [24]. This could be particularly relevant in the current treatment scenario for mHSPC where prospective studies are evaluating the efficacy of standard chemotherapy (i.e., docetaxel) and new generation hormonal treatment in combination with LHRH analogues [25]. Most of these aberrations have been detected in patients who progressed on ADT, and their clinical significance is both prognostic and predictive: prognostic, since their onset is associated to poor survival [26], and predictive, given the lower probability of response to other hormonal agents as a result of insensitivity and/or constitutively activation of the mutated *AR* [27]. However, to date, detection of *AR* gene alterations is not recommended in clinical practice, since it has not been prospectively validated for therapy selection [28]. Nevertheless, AR gain detected in plasma samples through NGS or digital-droplet polymerase chain reaction (PCR) has been proven to be associated with resistance to enzalutamide/abiraterone in both chemotherapy-naïve and post-docetaxel CRPC, with worse overall or progression-free survival and reduced PSA responses in mCRPC patients [29]. Patients with these gains seem to derive more benefit from taxane-based therapies as first- or second-line for mCRPC compared to hormonal agents [30,31,32]. Therefore, cell-free AR gains could represent an important predictive biomarker in patients previously exposed to AR pathway-targeting agents [33]. Similarly, expression of AR-V7 (assessed as AR-V7 mRNA and protein levels from tissue biopsies, circulating tumor cells [CTCs], and whole blood) is associated with resistance to AR-targeted therapies [22,34]. Additionally, a prospective study suggests CTC AR-V7 mRNA and protein expression are associated with worse PFS and OS in mCRPC patients treated with abiraterone and/or enzalutamide [35]. However, it is worth noting that not all mCRPC express CTCs; therefore, evaluation of AR-V7 might not always be possible in all patients. Moreover, a high CTC number per se is a prognostic factor associated with poor survival; therefore, it is difficult to ascertain the independent impact of AR-V7 on OS, especially considering that ARV-7 levels are related to the AR full length ones. These issues were sadly experienced during the ARMOR-III trail, a phase 3, randomized trial of galeterone versus enzalutamide in AR-V7-expressing mCRPC cancers. Overall, 953 men were prescreened for AR-V7: 323 (34%) had detectable CTCs, and 73/323 had AR-V7 mRNA with a prevalence of 8% (73/953). Of the 73 eligible patients, 38 were randomized to galeterone (*n* = 19) or enzalutamide (*n* = 19); 35 dropped out before randomization, consistently with worse outcome for high CTC/AR-V7 expressing mCRPC. This trial was prematurely closed based on interim evidence that the primary endpoint would not be met [36].

### 2.2. PTEN and PI3K-AKT Pathway

The phosphatase and TENsin homolog (PTEN) gene maps to chromosome 10 (10q23) and encodes for the homonym tumor suppressor protein, which acts as a phosphatase involved in physiological functions including embryonic development, stem cell growth and differentiation, cell adhesion, and migration [37]. It is mainly involved in the dephosphorylation of phosphatidylinositol 3,4,5-trisphosphate (PIP3) into phosphatidylinositol 4,5-bisphosphate (PIP2), with the opposite reaction being catalyzed by the PI3 kinase (PI3K). PTEN loss causes accumulation of PIP3 with an increase in phosphorylation of AKT and activation of its signaling pathway, ultimately causing unregulated cellular growth [38].

*PTEN* gene deletion is the most common alteration with an incidence higher than point mutations [39], while PTEN loss by IHC has been found in up to 40% of CRPC patients, being less frequent in localized disease [40]. Moreover, the absence of one of PTEN alleles causes insufficient quantity of protein to perform its biological task: This phenomenon is also known as haploinsufficiency [41]. Moreover, AR and PTEN pathways are regulated by reciprocal feedback [42], further supporting the relevance of the PTEN-PI3K-AKT pathway disruption in the development of prostate cancer. Interestingly, PTEN loss is also linked to high genomic instability (mainly aneuploidy) since its role as genomic integrity keeper is impaired [43].

PTEN loss has also been hugely investigated as a prognostic biomarker, and indeed it has been associated with higher risk of recurrence in localized prostate cancer after radical prostatectomy [44] and poor survival in metastatic patients [40,45,46]. PTEN loss by IHC has been extensively studied as predictive biomarker of response to hormonal treatment and chemotherapy agents. Two large retrospective studies showed indeed that PTEN loss (defined as less than 10% of cancer cells presenting positive staining) was associated with lack of response to abiraterone [40]; however, PTEN loss cancers had the same chance to respond to docetaxel as PTEN normal tumors [46]. PTEN expression was also studied prospectively as a predictive biomarker in mCRPC in a phase II randomized trial, which showed a longer rPFS in PTEN-loss tumors treated with ipatasertib, a small tyrosine-kinase inhibitor (TKI) against AKT [47]. A larger phase III study with ipatasertib (IPATential trial) has confirmed these results, although a different cut-off was used to define PTEN loss tumors (50% or more of the specimen’s tumor area having no detectable PTEN) [48]. Nevertheless, the benefit in terms of rPFS was also confirmed using more stringent cut-offs than the pre-specified 50%, consistently with what previously demonstrated in the phase II trial [49]. These sub-study analyses have also found a good concordance between PTEN status by IHC and by NGS. However, IHC is a cheaper method and the rate of quality control failure for tissue NGS in archival diagnostic samples must be taken in account when these methodologies are applied in clinical practice. Moreover, not always missense mutations translate into impactful alterations of a protein. Therefore, being the only prospectively validated test, IHC can be considered to date the gold standard for PTEN status.

Equally, PIK3CA-, PIK3CB-, and AKT-activating mutations have been studied as prognostic and predictive biomarkers in mCRPC [50]. As per PTEN loss, these activating mutations are associated with a poor prognosis, lack of response to hormonal agents, and might be able to predict response to AKT inhibition [51]. 

### 2.3. Homologous Recombination Deficiency

Homologous recombination deficiency (HRD) consists of the loss of ability of normal and tumor cells to repair double strand breaks (DSBs) that occur into the DNA [52]. Several proteins are deputed to maintain genome integrity through restoration of DSBs, from recognition of DNA damage to the joining of disrupted extremities [53]. In prostate cancer, genes encoding for these proteins have been found to be mutated in different percentages. In the work by Robinson et al., in which tissue specimens from 150 mCRPC patients were analyzed by whole-exome and transcriptome sequencing, *BRCA2* was the most commonly mutated HR gene (13.3%), followed by *ATM* (7.3%), *CDK12* (4.7%), and *BRCA1* (0.7%) [39]. Overall, these data were confirmed by the PROfound trial, a large phase III trial evaluating the efficacy of Olaparib in HRD mCRPC, whereas 27.9% of the 2792 successfully sequenced tumor specimens were found HR defective, confirming *BRCA2*, *CDK12,* and *ATM* as the three most frequent altered genes (harbored by 33.3, 23, and 22.2% of all the randomized patients, respectively) [54]. Some histological PCa variants, such as intraductal and cribriform, seem to be enriched for BRCA2 biallelic loss [55]; however, to date, no definite correlation between HRD mutations and morphological aspects could be made in PCa patients.

Compared to the metastatic disease, HRD incidence seems to be lower in localized prostate tumors (5–10%), showing an interesting correlation with Gleason score (GS) [56] and suggesting a role in progression and dissemination.

Most of the published works did not discriminate between germline and somatic HRD mutations [57]. In the work by Robinson et al., the prevalence of germline mutations is almost half of all detected mutations [39], but it must be noted that the percentage of germline alterations varies across ethnicities [57].

It is interesting to note that germline *BRCA1/2* and *ATM* mutations are characterized by worse prognosis in metastatic prostate cancer, whilst somatic mutations seem to not be [58,59]. Patients with germline *BRCA2* mutations have a risk as high as 20-fold of death due to prostate cancer compared to wild-type *BRCA2* population [60].

In addition, *CDK12* alterations, found in less than 4% of primary and in no more than 10% of metastatic prostate cancer [61], are associated with higher GS at diagnosis, immunosuppressive tumor microenvironment (TME), and worse prognosis [62], thus suggesting that CDK12 altered PCas have distinctive features from other genomic subtypes of prostate cancer.

The predictive role of mutations affecting HRD genes has been investigated in several clinical trials with PARP inhibitors in metastatic prostate cancer patients, starting from the assumption that PARP inhibition, by preventing single strand breaks (SSBs) repair, the cause death of cells who are unable to repair DSBs (also known as synthetic lethality). This “classic” model has been challenged during the last decade, since other mechanisms could contribute to cell death [63].

To date, among PARP inhibitors studied in prostate cancer, the main clinical data derives from the use of olaparib, niraparib, and rucaparib [64].

Olaparib has been tested in two phase II clinical trials, TOPARP-A and TOPARP-B. TOPARP-A, a small-size single arm study in molecularly unselected mCRPC patients, showed that olaparib was active in one third of them. The preplanned post hoc molecular analysis highlighted that 88% of HRD mutated patients obtained a response, paving the way for further research [65]. The phase II TOPARP-B trial was subsequently developed to confirm the activity of olaparib-at different doses, 300 vs. 400 mg QD-in HRD mutant mCRPC patients selected by targeted next-generation sequencing (NGS) panel on tissues samples, corroborating the predictive role of HRD for PARP inhibitors [66]. In the aforementioned phase III PROFound trial, HRD mCRPC patients who progressed to one hormonal agent (enzalutamide or abiraterone) were divided in two cohorts according to their mutational status—cohort A, patients with at least one alteration in *BRCA1*, *BRCA2,* or *ATM* genes, and cohort B, all the other patients—and then they were randomized between olaparib and the hormonal agent who was not previously received. This trial showed that olaparib prolongs PFS in cohort A and cohorts A + B, also prolonging OS in cohort A as reported by an interim analysis [54].

A recent gene-by-gene exploratory analysis of olaparib efficacy has been performed in PROFound trial patients. Despite limits related to the small numbers of patients in each subgroup, among the BRCA, ATM, CDK12, and CHEK2 cohorts, only patients with BRCA alterations maintain a statistically significant increase in PFS and OS when treated with olaparib [67]. 

Niraparib has been tested in the single-arm phase II GALAHAD trial, in which 300 mg QD of this drug was administered in HRD mCRPC patients; interestingly, a high response rate was achieved in *BRCA1/2*-mutated patients, but not in non-*BRCA1/2* ones, underlining that HRD mutations should be considered as a unicum in terms of predictiveness [68]. On the other hand, the rarity of non-BRCA and non-ATM mutations makes it difficult to assess their relevance.

Rucaparib has been tested in the single-arm phase II TRITON2 trial, in which pretreated mCRPC patients with deleterious germline or somatic alteration in *BRCA1, BRCA2,* or another prespecified DDR gene conferring sensitivity to PARP inhibition were enrolled [69]. In the independent radiology review (IRR) evaluable population, the overall response rate (ORR) and PSA response rate were 43.5 and 54.8%, respectively, confirming the class-effect of PARP inhibitors in this specific setting of mCRPC patients.

### 2.4. Mismatch Repair Deficiency

Base–base mismatches and insertion/deletion mispairs are generated during DNA replication and recombination as a consequence of “imperfect” activity of DNA polymerases, especially in the case of repeated sequence [70]. Repair of such errors is therefore essential for maintaining genomic stability since the accumulation of mismatches could lead to DNA disruption and cell death. In human biology, four genes (*MSH2*, *MSH6*, *MLH1*, *PMS2*) encode for homonym proteins which are responsible for repair machinery [71]. Mismatch repair deficiency (MMRd), which could be also caused by deficiency in other proteins such as exonuclease1 (EXO1) and polymerase ε (POLE), is a frequent alteration in human cancer [72].

Concerning prostate cancer, MMRd has been identified in about 5% of metastatic patients and the most frequently mutated genes are *MSH2* and *MSH6* [73]. Only a small percentage of MMR gene mutations are inherited (germline), with Lynch syndrome being rarely associated with prostate cancer, thus meaning that MMRd could be acquired by prostate cancer cells during disease evolution [74].

Absence of MMR mechanisms is a negative prognostic factor in metastatic prostate cancer, determining shorter OS in respect to mismatch repair proficient (MMRp) patients [75].

Similarly, to gastrointestinal and some gynecological cancers, MMRd has become an interesting predictive factor for immunotherapy also in prostate cancer, given the biological rationale of immune response against neoantigens which are overrepresented in microsatellite instability (MSI) high tumors as a consequence of increased tumor mutational burden (TMB) [76]. Antibodies against programmed death 1 (PD-1) receptor (anti-PD-1) have been firstly tested in non-molecularly selected docetaxel-refractory mCRPC patients, with modest results [77]. The correlation with response to anti-PD-1 depending on MMR status in mCRPC patients was demonstrated, with MMRd patients being the only group who reached objective responses and durable benefit from immunotherapy [78].

### 2.5. Tumor Microenvironment

The tumor microenvironment (TME) plays a fundamental role in human tumorigenesis. It consists of several immune and stromal cells: cancer associated fibroblasts (CAFs), endothelial cells, lymphocytes, macrophages, dendritic cells (DCs), and myeloid-derived suppressor cells (MDSCs), to mention a few [79]. The complex relationship among these cells and between TME cells and tumor cells has been under investigation to understand mechanisms of progression and immune evasion by tumoral cells, consequently influencing the response to therapies [80].

In prostate cancer, TME actively participates in different steps of tumorigenesis: during transformation, with CAFs replacing stromal smooth muscle cells; during tumoral growth, through angiogenesis upregulation; and at all stages, by regulation of immune cells [81].

Several biomarkers from TME have been evaluated in prostate cancer patients, most of them being soluble factors secreted by stromal/immune cells.

Cytokines—and particularly interleukins—are interesting prognostic (and potentially predictive) biomarkers in human cancer, including prostate cancer. Circulating levels of two proinflammatory cytokines, interleukin 6 (IL-6) and 8 (IL-8), have been found to be higher in hormone refractory and sensitive metastatic prostate cancer patients with poor prognosis, respectively [82,83]. IL-23, produced by MDSCs, could be implied in castration resistance through activation of AR pathway, thus suggesting a role for its blockade in this clinical setting [84].

Immune receptors, expressed on the surface of tumor-infiltrating immune cells, have been extensively studied in the latest years. The aforementioned PD-1 receptor is expressed on T-cells and leads to their exhaustion when it binds its ligand, PD-L1, mainly expressed in tumor cells [85]. Concerning prostate cancer, the expression of PD-1 and PD-L1 in localized tumors seems to be low [86], and this observation contrasts with previous studies on other cancer types. Moreover, PTEN loss is associated with a high expression of PD-L1, but not in prostate cancer [87], and neoadjuvant hormone therapy could reduce PD-L1 expression in localized prostate cancer [88]. However, other studies reported PD-L1 positivity in aggressive primary prostate carcinomas, also with a prognostic significance since it has been correlated to biochemical recurrence [89] and high expression in both preclinical and clinical models of enzalutamide-resistant prostate cancer [90].

The predictive role of PD-L1 is more complex to define, since its evaluation is strongly influenced by site (tumor vs. immune cells) and level of expression (high vs. low, depending on threshold value), also taking into account the multiplex interactions between TME and tumor cells [91]. Anti-PD-1 (nivolumab, pembrolizumab) and anti-PD-L1 (atezolizumab, avelumab, durvalumab) antibodies have been tested in phase I/II clinical trials in several settings of metastatic prostate cancer, with low response rates [91]. In fact, it seems clear that only some specific subgroups of prostate cancer patients—including those with PD-L1 positive and TMB high tumors—could really benefit from PD-(L)1 blockade, even if response to immunotherapy seems to be independent from PD-L1 expression [92]. In the CheckMate 650 trial, in which 90 mCRPC patients, divided into two cohorts based on previous exposure to chemotherapy, received nivolumab plus ipilimumab (anti-CTLA-4), a biomarker analysis was conducted [93]. Patients with a TMB higher than the median did perform better than those with a TMB lower in terms of the three main outcomes (PFS, ORR, OS), with similar results also according to PD-L1 expression levels (≥ or <1%). However, the variability of the methods used for TMB estimation and the absence of an established cut-off make the application of TMB still clinically impracticable. 

**Figure 1:**
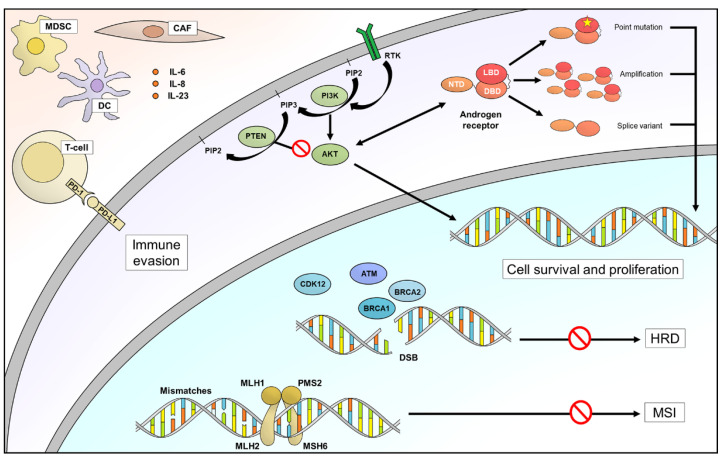
Predictive and prognostic biomarkers in prostate cancer. DC, dendritic cell; CAF, cancer associate fibroblast; MDSC, myeloid-derived suppressor cells; RTK, receptor tyrosine kinase; DSB, double strand break; HRD, homologous recombination deficiency; MSI, microsatellite instability.

An emerging biomarker in prostate cancer, expressed in both tumor and immune cells, is the ectoenzyme CD38. CD38 is responsible for non-canonical synthesis of adenosine, which inhibits antitumoral immunity by interacting with its receptor on several types of immune cells [94]. A recent work has highlighted an increase in CD38+ tumor-infiltrating immune cells in prostate cancer specimens after the onset of castration resistance, a high level being correlated to worse OS [95]. Similarly, a high expression in normal adjacent prostate epithelium of CD73, an ectoenzyme belonging to the same pathway and also responsible for adenosine synthesis, is associated with poor prognosis in prostate cancer patients [96]. Daratumumab, an anti-CD38 monoclonal antibody, is currently approved for heavily pretreated relapsed and refractory multiple myeloma patients, being a promising agent to be tested in refractory prostate cancer. Moreover, it could be hypothesized that CD38 expression levels are predictive for response to daratumumab in prostate cancer patients, as previously reported in refractory multiple myeloma [97] (Figure 1 and Table 1).

Overall, the discovery of biologically distinct subtypes has revolutionized the historical assumption that prostate cancer is a homogenous disease with an indolent behavior. There are indeed molecular characteristics that can significantly differentiate prostate cancers between patients and within the same patient overtime. However, all the aforementioned studies have used different methodologies and platforms for their own biomarker definition, and it is crucial to stress the differences between methods and tissue used. 

## 3. Tissue and Methods

### 3.1. The Tissue May Be the Issue

Screening approaches centered on specific biomarkers, such as prostate-specific antigen (PSA), have transformed the diagnosis and management of PCa, either in early or metastatic disease stage. Despite this, tissue specimens are crucial to formulate a final diagnosis. Clinicopathological variables that have historically guided patient stratification, such as Gleason grade and tumor stage, have been recently integrated with molecular assays, either in the form of as single gene testing or more extensive multigene profiling that may influence treatment decision making [28].

A key step when discussing tissue-based analyses, most importantly when these hold a prognostic and/or predictive impact, is to ensure that tissues are of adequate quality to be profiled. It is well known that the preanalytical phase in surgical pathology has a strong impact on the preservation of distinct types of molecules, hence pathology laboratories need to properly monitor the workflow of tissue biopsies and surgical specimens and to guarantee proper formalin fixation [98]. If transrectal or transperineal ultrasound-guided biopsies and prostatectomy specimens can be easily handled, on the other hand, the common bone metastatic deposits occurring in PCa patients may hamper the feasibility of molecular investigations. Indeed, in the latter scenario, the amount of tissue is often scant and requires decalcification, which can affect the quantity and quality of the nucleic acids available in the sample, reducing the chances of a successful test [99,100,101,102]. As reported by Zheng and colleagues, the failure rate using an NGS assay is higher in metastatic bone samples, and decalcification contributes to increasing failure [103]. However, the introduction of novel therapeutic approaches for metastatic prostate cancer requires a molecular profiling of these lesions to identify patients who may benefit from these potentially life-saving therapies.

Despite differences between the several guidelines currently available worldwide, testing for somatic and germline *BRCA1* and *BRCA2* alterations is becoming the minimum requirement for patients with metastatic prostate cancer [102]. Cooperation between clinicians and pathologists is paramount to ensure that appropriate bone decalcification methods are used to maximize nucleic acid preservation [104]. EDTA-based solutions have been shown to enable better performance compared to stronger decalcification procedures [99,100]. Indeed, EDTA-based decalcification delivers better results for DNA- and RNA-based NGS and in situ hybridization techniques than formic acid, suggesting that this reagent should be preferred [105]. When inquiring the biomarker data from the PROfound trial, for example, it is easy to realize that, overall, the success rate of the targeted sequencing from tissue specimens is 57% for archived samples (on a total of 4365 cases). This rate increases to 64% for newly collected biopsies, with the highest success (86.7%) for trephine bone marrow biopsies, appropriately profiled, although they represented only 15 of the 438 fresh collected tissue specimens (3.4%) [106].

### 3.2. Immunohistochemistry (IHC)

Immunohistochemical assays are relatively inexpensive and allow the detection of proteins by assessing both their localization and their heterogeneity of expression. The drawback relies on covering only a limited set of antigens, and, despite the possibility of multiplex-IHC is partially overcoming this issue [107], multiplex-IHC is yet to be part of our diagnostic armamentarium.

Although several biomarkers involved in cell proliferation, differentiation, apoptosis, and angiogenesis have been described for PCa, extensive profiling by immunohistochemistry does not play a leading role in the context of PCa pathology [108]. Conversely, immunohistochemical reactions support a proper diagnosis of PCa either in early disease (use of IHC staining for the basal cell layer in borderline cases when dealing with specimens presenting limited foci of atypical glands) [109], or in the advanced setting when the prostatic origin of a metastatic deposit needs to be confirmed [110].

In addition, in the context of metastatic disease, the evaluation of some markers may be of support. For instance, AR expression by IHC is feasible and reproducible. The binary output of AR expression assessment (present or absent in nuclei of tumor cells) increases the reproducibility of the test between laboratories [111]. AR expression assessment may have a role in clinical practice for de-escalating therapy with androgen receptor signaling inhibitors and for the evaluation of alternative treatment options in refractory metastatic castration-resistant prostate cancer, although detection of *AR* mutations and amplifications on liquid biopsies have been associated to outcomes in more recent years, as stated before [29].

PTEN protein expression can also be assessed by IHC on formalin-fixed paraffin-embedded tissues (FFPE), offering the possibility to identify PTEN loss due to other mechanisms than genomic deletion [112]. Given the cost-effectiveness of IHC testing, systematic PTEN IHC analysis could be easily implemented in the diagnostic workup of patients with metastatic prostate cancer. A validated IHC assay for PTEN evaluation based on a dichotomous scoring system for malignant glands, with cytoplasmic PTEN either present, or markedly decreased was proposed by Lotan et al. in 2011 [113]. Ferraldeschi and colleagues described, in the aforementioned work [40], a binary classification approach associated with clinical outcome in metastatic CRPC patients, in which cases were considered PTEN negative if they either showed a complete absence of PTEN staining or weak intensity staining compared with internal control in no more than 10% of cancer cells. The latter scoring system was also applied to show that response to taxane-based chemotherapy in metastatic CRPC is not affected by PTEN loss. In this scenario, PTEN intratumor heterogeneity was also taken into account, considering a case PTEN negative if any tumor area showed a complete absence of PTEN staining [46].

A recently published meta-analysis reported data on the evaluation of PD-L1 expression by IHC in prostate cancer. Positivity for PD-L1 was defined by applying different cut-off values, moving from considering PD-L1-positive a case with >50 of positively stained cells to define PD-L1 positivity as ≥1% of positively stained tumor cells. Dichotomization of PD-L1 expression based on median expression (high = above median, low = below median) was also evaluated [114]. In addition, several commercial anti PD-L1 antibody clones are available that may yield different results [115]. The phase 1b KEYNOTE-028 trial, designed to evaluate efficacy and safety of pembrolizumab for treatment of patients with PD-L1 positive advanced solid tumors (including 23 PD-L1 positive CRPC patients), defined PD- L1 positivity as the expression ≥1% in tumor or stroma cells using the 22C3 antibody (Merck) [116]. In the aforementioned KEYNOTE-199 study, Antonarakis and colleagues included three cohorts of patients with mCRPC to assess the antitumor activity and safety of pembrolizumab [77]; PD-L1 expression was evaluated in FFPE tumor specimens using the PD-L1 IHC 22C3 pharmDx assay (Agilent Technologies), and PD-L1 positivity was defined as a combined positive score (CPS) of ≥1, where CPS is the number of PD-L1 positive cells (tumor cells, lymphocytes, and macrophages) divided by the total number of tumor cells × 100.

Defective mismatch repair status can be indirectly determined by loss of mismatch repair protein expression by IHC. A cohort of 127 mCRPC specimens from the Royal Marsden Hospital was tested with antibodies against MSH2, MSH6, MLH1, and PMS2 proteins, as previously stated [75]. The stainings were classified as positive or negative using the College of American Pathologists criteria for biomarker evaluation in colorectal carcinomas: whenever tumor cells showed nuclear positivity, regardless of intensity, cases were considered positive, and cases with nuclear staining absent in tumor cells but present in background nonneoplastic tissue (internal control) were defined as negative [117]. A set of 316 prostate cancers on a tissue microarray (TMA) has been recently screened to determine MSI status by IHC: a loss of MMR protein was assumed if IHC staining was lacking in cancer cells, while clear-cut staining was present on adjacent stromal or inflammatory cells [118]. This analysis provided evidence that small tumor specimens can be suitable to predict the whole tumor MMR status, which is of paramount importance when prostate cancer core biopsies are scored for MSI to determine the potential eligibility of immune checkpoint inhibitors.

In localized and metastatic prostate cancer, different molecular subtypes may be identified [119,120,121,122,123]. In the field of PCa pathology, there is therefore a growing interest in patients’ stratification according to immunohistochemical surrogates mirroring PCa molecular subtypes identified by gene expression analyses, as already experienced for other disorders, such as breast cancer. Hammarsten and colleagues combined the assessment of PSA and Ki67 by IHC as surrogate markers for tumor cell differentiation and proliferation, thus classifying prostate cancer into subgroups of clinical significance [122]. On the other hand, Thysell and coworkers defined three molecular subtypes of bone metastases (MetA-C) with differences in gene expression pattern, morphology, and clinical behavior. In their work, the authors have suggested two different phenotype of PCa by combining PSA and Ki67 immunoreactivity, namely MetA-like (high PSA, low Ki67) and MetB-like (low PSA, high Ki67) [123].

The abovementioned selected IHC panel of tumor microenvironment biomarkers has also been tested to predict clinical recurrence in PCa [107]. The IHC panel included markers for CAFs (CD34, Cav-1, and αSMA), the vascular marker CD31, androgen receptor (AR), progesterone receptor (PR) and estrogen receptor (ER). Despite the small sample size and lack of validation in an independent patient cohort, this study demonstrated the feasibility of automated image analysis tools and digital pathology; this approach, used in support of traditional tissue analysis by IHC, could provide a more accurate quantification of proteins and bypass reproducibility problems in the evaluation of samples [107].

Despite the efforts to better characterize TME in prostate cancer, its complexity would require further investigation especially in the metastatic setting. An approach of great interest that could allow a detailed information is the CO-Detection by indEXing (CODEX) technology, recently applied in colon cancer on FFPE tissue specimens. CODEX is based on the use of oligonucleotide-conjugated antibodies for the simultaneous detection of 60 markers in a single tissue section and generates information on the distribution of different cellular phenotypes, while maintaining the morphological context [124,125,126]. CODEX application could be useful to characterize the composition, spatial organization, and functional immune status of the TME in FFPE metastatic PCa specimens.

### 3.3. Molecular Tests

Tissue-based molecular tests—such as Decipher [127], Oncotype Dx [128] and Prolaris [129], which are mRNA tests, and ProMark [130], which is a proteomic test—are currently extremely widespread even if not totally integrated in clinical practice, in early disease [131]. (Table 2). 

The American Society of Clinical Oncology (ASCO) has published a guideline to provide recommendations for the clinical use of the available tissue-based molecular tests in localized prostate cancer [132]. However, the use of commercially available molecular tests in routine clinical practice is not recommended. ASCO has emphasized that these tests may be considered when the assay result, studied in combination with routine clinical factors, clearly affects treatment decision making and influences patient management [133]. However, in the perspective of precision medicine, the identification of molecular features provides a tool for a better risk stratification. Jairath and colleagues recently published a systematic review of the evidence for the Decipher Genomic Classifier in prostate cancer, highlighting the usefulness of the test in defining which tumors are aggressive and in supporting the decision-making process for personalized treatment [134].

PCa is characterized by a wide clinical heterogeneity reflecting the molecular heterogeneity of the disease. A broad spectrum of recurrent genomic alterations has been identified, enabling the definition of distinct molecular subtypes of PCa based on molecular aberrations [135], as previously discussed in the “Predictive and prognostic markers” section of this review article. The genomic landscape of mCRPC has been well characterized, but the association of genomic features with patient clinical outcomes and histology or transcriptional pathway activity is not totally understood [136,137]. Therefore, PCa patient management still represents a challenge, particularly when dealing with intra-patient tumor heterogeneity and clonal evolution in metastatic disease. The European Society for Medical Oncology (ESMO) has recently presented recommendations about whether and how tumor multigene NGS could be performed to profile metastatic cancers [138]. Following these indications, in PCa patients, a multigene tumor NGS test should be performed on tumor samples to assess level I alterations. Hence, the mutational status of *BRCA1/2* in countries where PARP inhibitors are accessible for these patients should be provided. *PTEN* genomic alterations (deletions/mutations) and pathway aberrations (*PIK3CA*, *PIK3R1*, *AKT1*) could be added to the panel when treatments with AKT inhibitors are available. 

As part of the aforementioned IPATential trial [49], De Bono and colleagues evaluated indeed the concordance between PTEN status assessed by IHC and NGS, using the Foundation Medicine FoundationOne CDx NGS assay: The overall agreement was 85.5%. Among the samples with PTEN loss by NGS (*n* = 208), 190 (91.3%) were PTEN loss by IHC, while among the samples with PTEN loss by IHC (*n* = 247), 190 (76.9%) were PTEN loss by NGS. In addition, some patients with PTEN loss presented other PIK3CA and AKT gene alterations associated with worse prognosis [49].

In addition, multigene tumor NGS panels should include DNA repair genes and MSI signatures. Of note, the ESMO recommendations also stress the concept that larger panels require a substantial financial commitment, and they can only be used if they report accurate classification of alterations and if specific agreements are in place with payers for sustainability of costs [138].

The advantage of using NGS lies in the possibility of identifying useful information for patients care; however, this approach applied to PCa pathology still has some limitations. The analysis should be performed on FFPE tissue samples usually collected in routine diagnostics. Each assay usually has specific requirements in terms of the amount of material necessary to run the test and sometimes specimens may have nucleic acids of poor quality [139]. The scenario becomes even more complex when longitudinal disease monitoring is considered, aiming to study the progression of the disease and to associate the most effective therapy. Monitoring requires repetitive biopsies that expose the patient to invasive procedures in the attempt to obtain an adequate and suitable tissue sample [139]; however, using fresh collected specimens has the exquisite advantage to give an updated representation of the alterations acquired overtime by the disease. This is extremely relevant for activating mutation in *PIK3CA* and *AKT*, largely subclonal hence not present from diagnosis, which can predict response to AKT inhibition strategies [49]; or for the treatment emergent AR amplification and mutation that predict resistance to AR targeting agents [95].

Moreover, NGS approaches on recently acquired tissue are able to detect emerging *RB1* loss and *TP53* mutations, which associate with neuroendocrine phenotype, identifying treatment emergent neuroendocrine prostate cancer (t-NEPC) that still represent a challenge for clinicians from the treatment point of view [140].

Transcriptomic profiling may also play a role, beyond prognostic signatures for early disease. A recent analysis through PAM50 classifier on specimens from 160 patients enrolled in the phase III CHAARTED trial (ADT with or without docetaxel in mCSPC patients) highlighted that basal, luminal B, and luminal A signatures were present in 50, 48, and 2% of the specimens, respectively [141]; these percentages are different from those reported for localized PCa [142]. Interestingly, the luminal B signature was associated with poorer OS on ADT alone when compared to basal signature, together with OS improvement by adding docetaxel. However, to date, in the absence of validation studies and, most importantly, waiting for similar analysis from trials conducted in the same setting, this signature should not influence clinicians’ choice in treating mCSPC.

Brady and colleagues have recently proposed a new approach to study metastatic PCa by investigating the potential informativeness of the digital spatial profiling (DSP) technology, a new approach able to assess and quantify gene expression and protein abundance in spatially-distinct regions of FFPE tissue specimens [143]. The study cohort included 27 patients with refractory metastatic PCa, resulting in FFPE specimens from 52 soft tissues metastases and four bone metastases employed for the construction of TMAs. Serial sections of the TMA were used for histological analysis and simultaneously stained with fluorescently labeled antibodies specific for CD3 and CD45 markers, pan-cytokeratin for epithelial cells and a nuclear stain to facilitate the identification of tissue morphology for DSP; the DSP panel for mRNA analyses included probes for 2093 unique genes, comprising signatures of AR activity, neuroendocrine differentiation, proliferation, fibroblast growth factor (FGF), and mitogen activated protein kinase (MAPK) activity, loss of the retinoblastoma gene (RB1), and markers of cell types including macrophages, T cells and B cells. To assess protein expression, the antibody panel consisted of oligo-conjugated antibodies, including AR, synaptophysin and other 55 proteins of interest; following probe hybridization, UV cleavage, and barcode collection, gene expression was quantitated by Illumina sequencing (for protein) or by PCR amplification and Illumina sequencing (for RNA). All of the tases evaluated by DSP profiling had matched bulk tumor whole-transcriptome RNA-seq data to be compared with the DSP results. Starting from TMAs, the authors were therefore able to study heterogeneity, to identify distinct phenotypes, to assess several biomarkers associated with specific treatments, and to quantify the intratumoral immune cell composition useful to investigate the lack of responses to immune-based therapy observed in patients with metastatic PCa. The authors detected a high concordance in the intratumoral phenotypic makeup of the cases under investigation. In addition, an overall absence of immune cell infiltrates was reported in the majority of the metastases and high levels of expression of the immune checkpoint proteins B7-H3 and TIM-3 were identified. This last issue is of great interest if it is considered that target therapies against B7-H3 are currently under consideration in clinical trials for several solid tumors [144]. The specimens employed for these tests showed no age-related variation, indicating that this assay is suitable both for retrospective and prospective studies [143]. 

### 3.4. Blood-Based Tools

The employment of liquid biopsy is a valuable alternative to overcome some of the issues associated with the use of tissue specimens in studying and monitoring metastatic PCa patients.

Circulating tumor cells (CTCs), genetic materials such as cell-free RNA and DNA, as well as extracellular vesicles represent the analytes available in liquid biopsy [145]. They can provide a non-invasive summary of the total tumor burden of a patient and offer important data on therapeutic targets and mechanisms of drug resistance [146].

The main features of CTCs are rarity and heterogeneity: This is why separation and enrichment are challenging and represent the main limitation of this approach [147]. There are several sorting protocols that exploit different features of CTCs, such as volume, density and biomarkers. Although these methods are fast and offer not only the possibility of counting cells but also to analyze them after isolation, they have several limitations. For example, the CellSearch^®^ is an IVD-certified method in US for separation and counting of CTCs and it is based on the detection of epithelial cell adhesion molecule (EpCAM). Unfortunately, since CTCs are heterogeneous and undergo epithelial to mesenchymal transition (EMT) to escape the primary tumor, losing EpCAM and/or cytokeratin expression, this approach is not always ideal to succeed [146,147]. There are other commercially available platforms for CTCs isolation based on different technologies but despite the advantages of each one, there are also many drawbacks, including low cell viability or moderate sensitivity and doubts about accuracy in capturing viable CTCs [148]. Nevertheless, many studies have investigated the role of CTCs in PCa with respect to clinical utility. The number of CTCs has been correlated to therapeutic response and survival in metastatic PCa: Patients with shorter progression-free survival and overall survival have been shown to have higher CTC counts [146,147,149]. In addition, genomic analyses have been recently performed on CTCs, highlighting that CTCs derived from aggressive PCa show a high number of variants (single nucleotide variants and insertion/deletions) [147]. Genomic instability in CTCs has been correlated with aggressiveness in PCa, allowing to discriminate among metastatic PCa those cases prospectively stratified as resistant to therapy and with poor prognosis [149]. These studies have underlined the possibility to apply single-CTCs sequencing as a tool to noninvasively depict cancer heterogeneity.

Several studies have also investigated the role of circulating tumor DNA (ctDNA) in metastatic PCa patients. The analysis of ctDNA represents an additional low invasive and easily repeatable approach useful to monitor tumor progression and able to provide information on tumor molecular status and prognosis, complementing clinical data [150,151]. Moreover, quantitative studies on the changes in tumor fraction in serial samples treated with PARP inhibitors and taxanes have been associated with patients’ outcome and treatment responses [152]. ctDNA has been also used to identify genetic alterations since both somatic and germline mutations can be detected through ctDNA analyses. An important advantage of using ctDNA is the possibility of complementing somatic information from metastatic sites to investigate the mutational heterogeneity of the tumor by gaining a more reliable data compared to the information retrievable from a single tissue biopsy [153,154]. 

A recent genomic analysis of cfDNA in 3.334 advanced PCa patients has been reported showing that 94% of patients had detectable ctDNA [155]. Moreover, 837 patients in this analysis had both liquid and tissue (archival or metastatic) available for NGS. The authors demonstrated that comprehensive genomic profiling on ctDNA overall recapitulated the genomic landscape observed in tissue biopsies, with a high level of agreement in detection of some of *BRCA1/2* alterations. *BRCA1/2* were mutated in 8.8% of the analyzable cohort and the analysis identified a higher number of *BRCA* positive patient in liquid biopsies compared to tissue samples. This may be due to the fact that some patients may have gained somatic *BRCA1/2* alterations since archival tissue was collected were also identified. However, the discordance between tissue and liquid biopsy may also stem from the possibility that approximately 10% of men with advanced prostate cancer has clonal hematopoiesis (CHIP) interference in plasma cfDNA [156]. 

Moreover, the median tumor fraction in those samples was 7.5%; however, the threshold for detection of gene amplification in this analysis was ≥20%, meaning that information about amplification/deletion was possible in only 38% of the overall samples.

Another limitation for ctDNA analyses relates to the detection of larger genomic alterations. For example, durable responses to PARP inhibitors in prostate cancers are associated to homologous deletions of the *BRCA2* gene [157], but the rate of concordance between tissue and blood based NGS is definitely low for the detection of deletion/rearrangement for *BRCA2/1* and *ATM* (43%), in favor of the former [158]. This must be taken into account when choosing the analysis to be performed. 

Finally, extracellular vesicles (EVs), due to their specific loads such as proteins, mRNAs, miRNAs, lncRNAs, and lipids, exert important effects on cell signaling and tumor progression and are emerging as possible markers to monitor PCa progression and metastasis [159,160]. In addition, they are also indicators of therapy response in many cancer types, including PCa [161]. Proteomic studies performed in PCa cell lines and patients showed that EVs are a source of intracellular proteins and may be useful to improve PCa diagnosis [162]. Currently, the major limitation in the use of EVs in clinical practice is the lack of a standardized method for the detection and isolation of EVs, due to their small sizes and low densities. Although many technologies are available, the yield is low, and samples are not pure but contain contaminating proteins and reagents [159,161]. The selection of the isolation method to use is related to the downstream application. Recently, a new method based on imaging mass cytometry has been proposed to study and characterize large EVs in parallel to CTCs in metastatic PCa through a multiplexed protein profiling [162]. However, this is a preliminary approach that needs further investigation. It is also interesting to note that the EVs analysis could improve the detection of specific alterations such as AR variants, being a potentially useful tool in the next future in PCa biomarker research [163].

## 4. Conclusions

Advanced prostate cancer is still an incurable disease; however, the identification of molecular factors predicting prognosis and response to specific treatments have yielded new hope in this scenario. It remains, however, debatable which biological specimen to test, since the analyses on both tissue-primary tumors and/or metastases-and blood samples have several intrinsic characteristics and limitations. Using tissue samples gives the exquisite advantage to perform complemental analyses to genomic sequencing, such as immunohistochemical and TME studies. On the other hand, plasma or blood derivate are easy to obtain and allow serial specimens collection. Preservation of archival tissue, feasibility of fresh tissue biopsy, and adequate tumor fraction in plasma remain the big limitations of these tests. In the absence of a unique test or tissue that can provide all the information needed to give a correct representation of patients’ disease biology, ideally, both tumor tissue and blood should be interrogated to study predictive and prognostic factors in mCRPC, and most importantly to investigate new mechanisms of resistance to the approved treatments in this setting, depending on the clinical history of the patient.

## Figures and Tables

**Table 1:** Predictive and prognostic biomarkers in mCRPC. mCRPC: metastatic castration-resistant prostate cancer.

Class	Alteration/Level of Expression	Prognostic Impact	Prediction of Response
Androgen receptor (AR)	AR point mutations	Poor survival [26]	Lower probability of response to hormonal agents [27]
	AR amplifications		
	AR variants		
PTEN and PI3K-AKT pathway	PTEN loss	Poor survival [40,45,46]	Potentially predictive for AKT inhibitors [47]
Homologous recombination deficiency (HRD)	BRCA 1 or 2 mutations	Worse prognosis in case of germline alterations [58,59]	Predictive for PARP inhibitors [64,65,66,67,68]
	ATM mutations		
	CDK12 mutations	Worse prognosis [62]	
Mismatch repair deficiency (MMRd)	MSH2 and/or MSH6 loss	Shorter OS [75]	Predictive for PD-1 inhibitors [78]
Tumor microenvironment	IL-6 and IL-8	Poor prognosis [82,83]	Unknown
	PD-L1	Correlated to biochemical recurrence [89]	Potentially predictive for PD-(L)1 inhibitors [91,92]
	CD38	Shorter OS [95]	Potentially predictive for CD38 inhibitors [97]

**Table 2:** Tissue-based molecular tests in prostate cancer.

Test	Company	Sample	Type of Methodology	Output	Intended Use
Decipher [127]	Decipher Biosciences	FFPE tissue from prostate biopsy or prostate tissue after radical prostatectomy	mRNA expression of 22 genes	Decipher score (range: 0–1)	On biopsy: to stratify PCa patients for surveillance or treatment. On surgical tissue: to guide if surveillance, adjuvant, or salvage therapy can be assured.
OncotypeDx [128]	Genomic Health	Tumor tissue from original biopsy	mRNA expression of 17 genes (12 cancer-related genes plus 5 reference genes)	GPS score (0–100)	To assess who may benefit from surveillance or treatment.
Prolaris [129]	Myriad Genetics Inc.	FFPE tissue from prostate biopsy or radical prostatectomy	mRNA expression of 31 cell-cycle progression genes	CCP score (range: 0–6)	To define a 10-year risk of metastasis after treatment, and disease-specific mortality under conservative management.
ProMark [130]	Metamark	FFPE tissue biopsy	Quantitative expression of 8 proteins	ProMark score (range: 0–100)	To determine the aggressiveness of PCa.

Abbreviations: FFPE, formalin-fixed paraffin-embedded; GPS, Genomic Prostate Score; CCP, cell cycle progression.
